# Inorganic Nanocarriers for Encapsulation of Natural Antimicrobial Compounds for Potential Food Packaging Application: A Comparative Study

**DOI:** 10.3390/nano11020379

**Published:** 2021-02-02

**Authors:** Tina Gulin-Sarfraz, Georgios N. Kalantzopoulos, Marit Kvalvåg Pettersen, Anette Wold Åsli, Ingunn Tho, Lars Axelsson, Jawad Sarfraz

**Affiliations:** 1 Nofima-Norwegian Institute of Food, Fisheries and Aquaculture Research, P.O. Box 210, NO-1431 Ås, Norway; marit.kvalvag.pettersen@nofima.no (M.K.P.); anette.wold.asli@nofima.no (A.W.Å.); lars.axelsson@nofima.no (L.A.); 2 Center for Materials Science and Nanotechnology (SMN), Department of Chemistry, University of Oslo, P.O. Box 1033, Blindern, NO-0315 Oslo, Norway; georgios.kalantzopoulos@kjemi.uio.no; 3 Department of Pharmacy, University of Oslo, P.O. Box 1068, Blindern, NO-0316 Oslo, Norway; ingunn.tho@farmasi.uio.no

**Keywords:** porous silica particles, nanoclays, halloysite nanotubes, encapsulation, active, antimicrobial, thymol, curcumin

## Abstract

Design and development of novel inorganic nanocarriers for encapsulation of natural antimicrobial substances for food packaging applications have received great interest during the last years. Natural nanoclays are the most investigated nanocarriers and recently interest has also grown in the synthetically produced porous silica particles. However, these different carrier matrices have not been compared in terms of their loading capability and subsequent release. In this study, the feasibility of porous silica particles (with different pore structures and/or surface functionalities) and commercially available nanoclays were evaluated as encapsulation matrices. Two well-studied antimicrobial substances, thymol and curcumin, were chosen as volatile and non-volatile model compounds, respectively. The encapsulation efficiency, and the subsequent dispersibility and release, of these substances differed significantly among the nanocarriers. Encapsulation of the volatile compound highly depends on the inner surface area, i.e., the protective pore environment, and an optimal nanocarrier can protect the encapsulated thymol from volatilization. For the non-volatile compound, only the release rate and dispersibility are affected by the pore structure. Further, water-activated release of the volatile compound was demonstrated and exhibited good antimicrobial efficacy in the vapor phase against *Staphylococcus aureus*. This comparative study can provide a base for selecting the right nanocarrier aimed at a specific food packaging application. No nanocarrier can be considered as a universally applicable one.

## 1. Introduction

Food packaging provides many functions such as containment, protection, and preservation of the product. It also serves as a communication and marketing tool to attract customers. In recent years, a great deal of research effort has been put to improving the preservation ability of the food packaging in the form of active packaging systems [1,2,3,4,5]. Active packaging has been defined by the European Commission regulation (EC) No 450/2009 as packaging systems that are designed to interact with the packed food by either releasing or scavenging substances into or from the food or the environment surrounding the food [6]. The active packaging systems have been frequently reported in the literature with antioxidant and antimicrobial functionality to delay the oxidative and microbial spoilage of food, thus resulting in their improved shelf life [7,8]. More recently, there has been an increasing interest in the natural plant-based materials including essential oils (EOs) and their major components due to their reported antibacterial, antioxidant, and antifungal properties [9,10,11,12,13]. EOs such as cilantro, cinnamon, oregano, thyme, lemon grass, and their active components, for instance, linalool, cinnamaldehyde, carvacrol, thymol, and citral, may substitute for some of the synthetic additives. Furthermore, several of the EOs are classified as “Generally Recognized as Safe” (GRAS) by the US Food and Drug Administration (FDA) [14] and have been in use for a long time as flavoring agents for food.

The main disadvantages of essential oils and their major components are their volatility and tendency to oxidize, as well as their high intensity in odor [15]. This limits their processing to mainly cold-coating technologies [16,17]. To overcome this challenge, nanocarriers have been validated for encapsulation of these compounds [18,19,20]. Encapsulation allows relatively high-temperature processing [19]. Further, a sustained release can be obtained that may prolong the antimicrobial effect [20], while the negative organoleptic effect on the food products can be minimized [18]. Nanocarriers are here defined as materials with any external dimension in the nanoscale (1–100 nm) or those having internal or surface structures in the nanoscale [21]. Recent studies have explored the potential of using inorganic nanocarriers such as nanoclays for providing a robust protective environment for the active compounds. Nanoclays are natural nanomaterials and are the most commercially applied nanomaterials for food packaging applications [22]. Halloysite nanotube (HNT) is one type of nanoclay, consisting of two-layered aluminosilicate clay minerals with a hollow tubular structure [23]. It has been reported that HNTs can be successfully loaded with various compounds, such as insulin [24] and ibuprofen [25] for biomedical applications, and EO components (carvacrol and thymol) [26,27,28,29] for food packaging applications. These studies have shown the potential of HNTs to increase the solubility, the thermal and chemical stability, as well as the release rate of the active compounds.

Another nanomaterial that has been excessively studied for applications in the biomedical field, but has only recently gained attention as an antimicrobial carrier for food packaging applications, is porous silica. Porous silica nanoparticles offer many unique advantages. They can be tailor-made by tuning their size, morphology, pore size, and pore structure. Silica nanoparticles are also one of the most biocompatible nanocarriers, as synthetic amorphous silica is approved as food additive both by the European Food Safety Authority (EFSA) as E 551 [30,31] and by the FDA [32]. Additionally, silica nanoparticles can degrade into non-toxic silicic acid [33]. Recent studies have evaluated silica particles to function as carriers for EO components for packaging applications, either by physically loading the active compounds into the pore structure [34,35] or by covalently anchoring them to the silica surface [36]. In the latter case, the antimicrobial effect is based upon direct contact between the silica particles and the bacteria, and not by release of the compounds.

Even though nanoclays, especially the HNTs, have been investigated for encapsulating active compounds for food packaging applications during the last years, and recently also porous silica particles have found their way into the field, these different carrier matrices have not been compared in terms of their properties affecting loading capability and subsequent release. Therefore, in this study we intend to investigate and discuss benefits and drawbacks between nanocarriers possessing different morphology, size, specific surface area, and pore structure. To study the performance of the different nanocarriers, we selected thymol and curcumin as examples of volatile and non-volatile active components, respectively. Both these compounds are well-studied for their antimicrobial and antioxidant properties [37,38,39,40,41] and have been frequently studied for antimicrobial food packaging applications [42,43,44,45,46,47,48,49,50]. Curcumin is approved as food additive (E 100) by the EFSA [51,52] and by the FDA as GRAS [53]. Thymol is also listed as a food additive by the FDA [54], and thyme oil (in which thymol is a major component) is as well recognized as a GRAS essential oil [14]. Here we compared commercially available inorganic nanocarriers with synthesized porous silica particles in terms of encapsulation efficiency, release of the active components, and the resulting antimicrobial effect.

## 2. Materials and Methods

### 2.1. Reagents and Materials

All chemicals used for the study were of analytical grade; hexadecylamine (90%), tetraethyl orthosilicate (TEOS, ≥98%), 3-aminopropyl-triethoxysilane (APTES, 99%), triethoxy-phenylsilane (PhTES, 98%), 2-propanol (≥99.5%), ammonium hydroxide (28–30% NH_3_), hydrochloric acid (HCl, 32%), cyclohexane, thymol (≥99.5%), curcumin (≥80%), fumed silica (FS), halloysite nanotubes (HNT), montmorillonite (MM) K10, polyethylene glycol (PEG) 6000 (M_n_ 5000–7000) and 4-(2-hydroxyethyl)piperazine-1-ethanesulfonic acid (Hepes) were purchased from Sigma-Aldrich (Merck KGaA). Tween 80 was obtained from VWR (Avantor), and absolute ethanol from antibac.

### 2.2. Synthesis of the Porous Silica Particles (SPs)

The SP-A, SP-B, and SP-C were synthesized by modifying a procedure reported by D. Kumar et al. [55]. Two grams hexadecylamine was dissolved in 200 mL isopropanol and 180 mL milli-Q (18.2 MΩ cm) water by magnetic stirring under slight heating. Thereafter, 2.7 mL NH_3_ (28–30 wt%) was added. For the synthesis of the **SP-A**, tetraethyl orthosilicate (TEOS) was used as silica source. Thus, 11.6 mL TEOS was drop-wise added under stirring to the solution. To create a hydrophobized particle, the **SP-B**, approximately 10 mol% of TEOS was exchanged to PhTES. Thus, 10.4 mL TEOS was pre-mixed with 1.3 mL PhTES, and carefully added to the synthesis solution. To prepare a particle with reduced size, the **SP-C**, 5 mol% TEOS was substituted with APTES. Here, 11 mL TEOS were mixed with 0.6 mL APTES and added to the synthesis solution. The synthesis solutions were left for overnight reaction under stirring, whereafter the particles were separated by centrifugation. The surfactant template was removed by extraction two times for 1 h in slightly acidic ethanol (0.1 M HCl). During extraction, the solutions were stirred and carefully shaken, with no sonication involved, to preserve the porous structure of the particles. Finally, the particles were washed with ethanol, centrifuged, and vacuum-dried at room temperature.

### 2.3. Nanocarrier Characterization Methods

Characterization of the size and morphology of the nanocarriers by scanning electron microscopy (SEM): SEM imaging was performed with a Hitachi SU8230 microscope (Hitachi, Tokyo, Japan), operated at 2 kV, and a Zeiss EVO 50 EP microscope (Zeiss, Oberkochen, Germany) operated at 15 kV.

Determination of the net surface charge of the nanocarriers: Zeta potential measurements were carried out to determine the net surface charge of the nanocarriers. Dispersions of 0.5 mg/mL nanocarriers in 25 mM Hepes buffer at pH 7 was measured on a Malvern Zetasizer Nano ZS instrument (Malvern Panalytical, Malvern, United Kingdom), using Malvern’s dip cell kit.

Nitrogen physisorption to determine the specific surface area (SSA), C-value, pore size, and pore volume of the nanocarriers: Nitrogen sorption measurements were performed on a BelSorp mini II instrument (MicrotracBEL Corp., Osaka, Japan) at 77 K. In each experiment approximately 40 mg of material was weighted into a 9.001 cm^3^ glass cell. The samples were pre-treated with annealing under dynamic vacuum for 4 h at 80 °C. The total SSA was extracted from the nitrogen adsorption isotherms via the Brunauer–Emmett–Teller (BET) method according to the literature [56]. Non-local density functional theory (NLDFT) calculations of the pore size distribution (PSD) were performed using the commercial BELMaster software (MicrotracBEL Corp., Osaka, Japan). The NLDFT calculation method was applied on the adsorption branch using the nitrogen physisorption data collected at 77 K. For the calculations, a cylindrical pore model was assumed.

### 2.4. Loading of the Active Components to the Nanocarriers

Prior to the loading, the nanocarriers were vacuum-dried at 80 °C for at least 4 h. The active substance (curcumin or thymol) was dissolved in cyclohexane by sonication, and the nanocarrier was added. To obtain the maximum loading, an excessive amount of the active compound, with a weight-ratio of 1.5:1 (active compound and nanocarrier), was used. The suspension was sonicated and vortexed repeatedly, and subsequently left for stirring for 20 h. The loaded nanocarriers were separated by centrifugation, washed with cyclohexane, and finally vacuum-dried at room temperature.

### 2.5. PEG-Adsorption on the Loaded Nanocarriers

The hydrophobic curcumin-loaded nanocarriers were coated with a PEG-6000 polymer to improve their dispersibility in aqueous solvent. The PEG-polymer was dissolved in Hepes at a concentration of 10 mg/mL. The loaded nanocarrier was first dispersed in Hepes buffer (25 mM, pH 7) by vortex and sonication, and then a 100 wt% amount of PEG-polymer was added to the nanocarrier drop-wise under sonication. The dispersion was left to react for 3 h under stirring, with several cycles of sonication and vortex. The coated nanocarriers were separated by centrifugation, washed with water to remove excess PEG-polymer, and vacuum-dried at room temperature.

### 2.6. Determination of the Loaded Amount of Active Components into the Nanocarriers

The loaded amount of curcumin or thymol inside the nanocarriers was validated by elution of the substances in ethanol. Loaded nanocarriers, at a concentration of 0.4 mg/mL in ethanol, were repeatedly stirred, vortexed, and sonicated for 2 h. Afterwards, the nanocarriers were centrifuged and the supernatants were collected. The absorbance of the supernatants from thymol-loaded nanocarriers was measured on an Agilent 8453 Ultraviolet-visible (UV-Vis) spectrophotometer (Agilent Technologies, California, USA), at a wavelength of 279 nm. The supernatants from curcumin-loaded nanocarriers were diluted 10 times with ethanol, and the absorbance was measured at 425 nm on a Synergy H1 plate reader (BioTek, Winooski, USA). The loaded amounts of active substances were determined by standard curves made from the free substances in ethanol at the specific wavelengths and with the respective instruments.

### 2.7. Evaluation of the Release and/or Volatilization of Active Components from the Nanocarriers

The release of the loaded compounds from the nanocarriers was studied in food simulant (20% ethanol) in line with the Official journal of the European Union regulations on food simulants [57]. Separate samples were prepared for each time point, in duplicates.

Thymol-loaded nanocarriers were placed in food simulant at a concentration of 0.5 mg/mL. At defined time points the nanocarriers were separated by centrifugation, and the supernatant was collected and measured at 279 nm on the Agilent 8453 UV-Vis spectrophotometer. The amount of thymol released was assessed with a standard curve made in the same solvent at the specific wavelength. The volatilization of the released thymol from the supernatant (food simulant) was evaluated by leaving the holders of the supernatants open under room conditions, and at defined time points measuring the thymol left in the solution. Separate holders were also used here for each time point. At longer time points, a small amount of the solvent was evaporating, which was remedied by adding fresh solvent. Further, the volatility of free and encapsulated thymol (dry-loaded nanocarriers) was studied by leaving small tubes, containing either free thymol or thymol-loaded nanocarriers, open in normal room conditions or under vacuum at room temperature. At specific time points the tubes were collected, and a known amount of ethanol was added. The remaining thymol was thus dissolved and measured on the UV-Vis spectrophotometer at 279 nm.

For the curcumin-loaded nanocarriers, a concentration of 0.05 mg/mL in food simulant was used to study the release. At specific time points the nanocarriers were separated by centrifugation. Due to the hydrophobicity of curcumin, the supernatants could not successfully be measured by UV-Vis. Instead, the nanocarriers were collected, dried, and a known amount was eluted in ethanol and measured at 425 nm on the Synergy H1 plate reader. The remaining amount of curcumin in the nanocarriers was determined by using a standard curve of curcumin in ethanol. For the PEG-coated curcumin-loaded nanocarriers the release was also studied in bacteria culture media (Tryptic Soy Broth, TSB, with addition of 0.5 wt% Tween80). Here, a concentration of 0.3 mg/mL was used. At defined time points, the nanocarriers were separated by centrifugation, and the supernatants were diluted (1:5) prior to the absorbance measurements at 425 nm. A standard curve of curcumin in food simulant was utilized for determining the amount of curcumin released.

### 2.8. Determination of the Antimicrobial Response of the Released Active Compound

The antimicrobial response of the volatile thymol compound released from the nanocarrier was evaluated on the growth of *Staphylococcus aureus* ATCC 6538 by the time-to-detection method and the vapor diffusion method.

**Figure 1:**
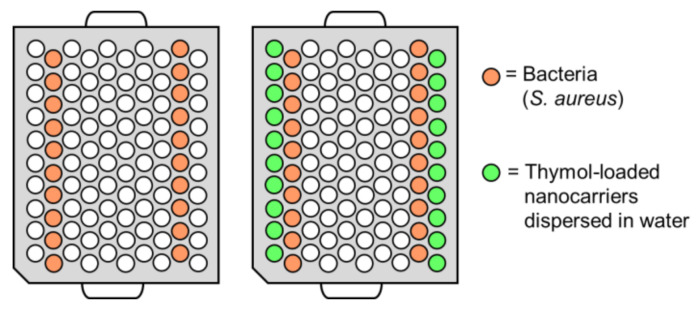
Illustration of the sample layout on the honeycomb-structured bioscreen plates.

For the time-to-detection method, thymol-loaded nanocarriers were dispersed in water and placed in a 100-well plate next to bacteria samples, as illustrated in Figure 1. The bacteria cells were grown in TSB and diluted to approximately 1 × 10^5^ cells/mL prior to the experiment. A volume of 200 µL of the particle dispersion or bacteria suspension was added to each well (Figure 1). Control bacteria samples were placed on a separate plate. A high concentration of 900 µg/mL loaded thymol (2.7 mg/mL SP-A loaded with 34 wt% thymol) was chosen to ensure that a sufficient amount of volatilized thymol reached the bacteria samples, since the plates used in this method were not air-tight. The growth curves of the bacteria were followed by absorbance measurements on a Bioscreen C instrument (Oy Growth Curves Ab, Ltd., Helsinki, Finland).

**Figure 2:**
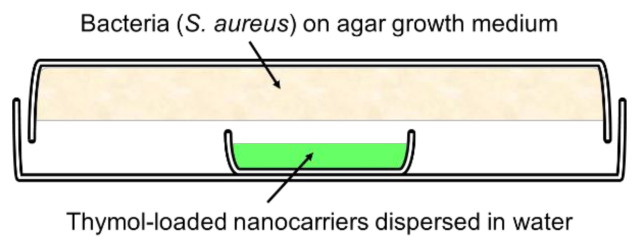
Schematic image of the experimental set-up of the vapor diffusion method.

To determine the antimicrobial effect by vapor diffusion method, a bacterial suspension of 200 µL, containing 1000 colony-forming units (CFUs)/mL *S. aureus*, was spread on tryptic soya agar (TSA, Oxoid) plates (20 cm in diameter). Thymol-loaded nanocarriers were dispersed in water and 3 mL of the dispersions were added to small lids, as schematically illustrated in Figure 2. Two concentrations were used: 100 µg/mL loaded thymol (0.3 mg/mL SP-A loaded with 34 wt% thymol) and 300 µg/mL loaded thymol (0.9 mg/mL SP-A). The agar plates were placed upside down with no direct contact between the agar and the nanocarrier dispersions. The plates were sealed with parafilm to keep them air-tight. The plates were incubated at 37 °C for 48 h, whereafter the CFUs were counted.

The macrodilution method was utilized to examine the antimicrobial effect of curcumin on *S. aureus*. The detergent Tween 80 was used to enhance the solubility of curcumin. An amount of either 50 µg/mL or 200 µg/mL curcumin was either dissolved directly in the bacteria culture media (TSB, with addition of 0.5 wt% Tween80), or pre-dissolved in 10 wt% Tween80 with further dilution with TSB to reach the required concentrations of curcumin and Tween80. Tubes containing 4 mL of the curcumin solutions and 1 × 10^3^ or 1 × 10^5^ bacteria cells/mL were incubated at 37 °C for 4, 24, and 48 h. After incubation, the spread plate technique was used to determine the viable bacteria number by counting the CFUs.

## 3. Results and Discussion

**Figure 3:**
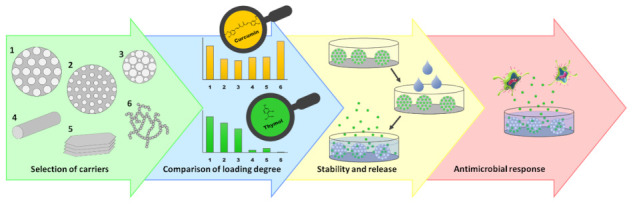
Schematic overview of the study.

Porous silica particles of various size and morphology, as well as commercially available fumed silica (FS) and nanoclays (montmorillonite (MM) and HNTs) were compared in terms of their feasibility to be used as carriers for natural active compounds. As natural active compounds, curcumin was chosen as model cargo for a hydrophobic non-volatile compound, and thymol was selected as model cargo for a volatile compound. Both of these compounds have earlier been reported to have antimicrobial and antioxidant properties [37,38,39,40,41] and have potential to be used for active food packaging applications [42,43,44,45,46,47,48,49,50]. Encapsulation efficiency, improvements (solubility and stability) of the active compounds by encapsulation, subsequent release, and antimicrobial properties were studied, as schematically illustrated in Figure 3.

### 3.1. Design and Characterization of the Nanocarriers for the Active Compounds

**Figure 4:**
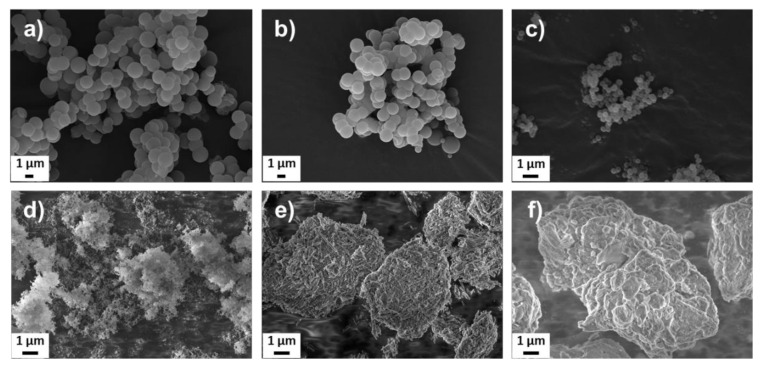
Scanning electron microscopy images of the different nanocarriers: (**a**) SP-A, (**b**) SP-B, (**c**) SP-C, (**d**) FS, (**e**) HNT, and (**f**) MM.

The porous silica particles (SPs) were produced with modifications of the synthesis reported by Kumar et al. [55], which gives highly porous particles composed of a random close-packing of smaller primary nanoparticles. To alter the size, morphology, and surface chemistry of the particles, co-condensation of siloxane and organosiloxane precursors were employed. The scanning electron microscopy (SEM) images shown in Figure 4a–c, reveal spherical particles on the micron and nanoscale; SP-A and SP-B sized around 1500 nm and SP-C around 250 nm. The SP-A were synthesized by only siloxane precursor to obtain a purely siliceous particle. The SP-B were co-condensed using a phenylsilane to create a suitable environment for loading of hydrophobic compounds. Co-condensation with aminosilane is known to affect the size [58] and pore structure [59] of porous silica particles and was thus used for SP-C to decrease the particle size, while the pore size was increased. To obtain a porous network, hexadecylamine was added as template molecules to the synthesis solution. The template molecules must carefully be removed to preserve the porous structure and not cause aggregation of the particles, at the same time as properly removing all the template. Here, the template was extracted in slightly acidic ethanol with careful mixing, as was previously shown to be the optimal method for these specific porous particles [58].

The SEM images of the commercial counterparts, FS and nanoclays, are presented in Figure 4d–f. The FS, formed by small primary nanoparticles branched into long chain-like aggregates, are reported by the manufacturer to have an average size of 200–300 nm. HNTs have a hollow tubular-like structure with reported inner and outer diameters of about 10–15 nm and 50 nm, respectively, and length of 600–1000 nm [23]. MMs are comprised of stacked nanolayers of varying sizes. The reported thickness of one layer is about 1 nm [60].

All the carrier materials compared in this study had a similar overall net surface charge (zeta potential, measured in Hepes buffer at pH 7), as listed in Table 1. The magnitude of the zeta potential gives an indication about the electrostatic stability of the particles in solution at a specific pH. As a general rule, a zeta potential of ±30 mV is considered as electrostatically stable. However, this is only relevant when charge repulsion between the particles is the only means of stabilization. Other factors also affect the dispersibility, and nanoparticles can be further surface coated to provide steric stabilization. The SP-A, FS, and HNT measured a similar zeta potential (−27 mV, −28 mV, and −31 mV) due to the same outer surface groups. It can be noted here that the HNTs consisted of two-layered aluminosilicate clay minerals and were thus positively charged inside the tubular pore structure. The MM, which also comprised layered aluminosilicate, had a slightly higher zeta potential of −21 mV. The phenyl- and amino-co-condensed silica particles (SP-B and SP-C) had also higher zeta potentials of −22 mV and −19 mV due to the different surface groups, compared to SP-A.

The porosity and the high surface area of the particles were assessed by nitrogen physisorption measurements. The specific surface area (Brunauer–Emmet–Teller, BET) was determined to be 720 m^2^/g for SP-A, 830 m^2^/g for SP-B, and 420 m^2^/g for SP-C. As a comparison, HNTs were also measured and resulted in a BET area of about 40 m^2^/g. The reported surface area for the HNTs by the manufacturer was 64 m^2^/g. Prior to the nitrogen sorption measurements in this study, all the samples were degassed under 80 °C only, so as not to affect the organic surface groups on the SP-B and SP-C samples. This might be the reason for the lower specific surface area for the HNTs in our study. However, the degassing conditions were identical for all samples for comparison purposes. Further, the reported surface area for the FS used in this study was about 200 m^2^/g and for MM 220–270 m^2^/g.

**Figure 5:**
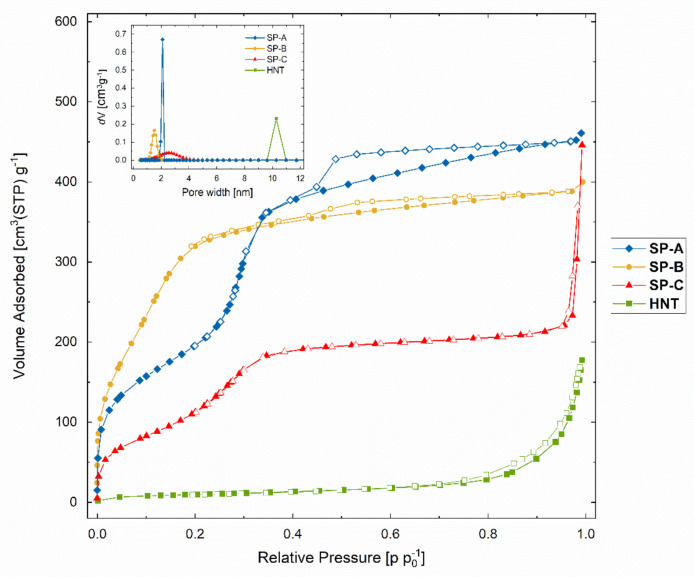
Nitrogen physisorption isotherms and pore size distribution plots (inset) of the different porous silica particles (SP-A, SP-B, SP-C) in comparison to halloysite nanotubes (HNTs). The adsorption branch of the isotherms is illustrated with full symbols and the desorption branch with hollow ones.

The nitrogen physisorption isotherms are presented in Figure 5. An adsorption isotherm is the relation between the amount of gas (nitrogen) adsorbed and the equilibrium pressure of the gas, at a constant temperature [61]. The International Union of Pure and Applied Chemistry (IUPAC) classified six types of isotherms and six types of related hysteresis loops, which are closely associated to certain pore structures and underlying adsorption mechanisms. Furthermore, IUPAC recommends to distinguish pore sizes into macropores (>50 nm), mesopores (2 to 50 nm), and micropores (up to 2 nm) [62]. Both SP-A and SP-C had isotherms of type IV, which is characteristic of mesoporous materials. On the contrary, the main capillary condensation step was clearly altered toward lower relative pressures for the SP-B, thus shifting toward a type I(b) isotherm (Figure 5), typical for microporous solids. The hysteresis loop (H4) indicated narrow pores, which was confirmed with a high value of the constant C (160) in the BET equation that is also generally associated with the filling of narrow micropores (Table 1) [61]. According to the BET theory, for mesoporous materials the C-value is related exponentially to the energy of the first layer adsorption. However, that is not applicable to microporous materials where the C-value rather gives an indication of the shape of the isotherm. The C-value for SP-A and HNTs were both about 60, due to their similar surface chemistry. The surface amino groups on the SP-C probably affected the affinity between the nitrogen molecules and the particle surface, and thus resulted in a lower C-value of 35 (Table 1).

To further compare the pore size distribution and pore volume of the particles, the non-localized density functional theory (NL-DFT) was applied for the analysis. Since the pore structures of the particles and HNTs differed and did not fall under a specific category of pore shape, a cylindrical pore model was assumed for the calculations. The pore size distributions shown in the inset graph in Figure 5 are presented for comparison purposes between the different materials and to confirm the micropore size of the SP-B. The calculated value for the pore size was 1.5 nm with a pore volume of 0.7 cm^3^/g. The pore size for the SP-A was slightly over 2 nm and pore volume 0.8 cm^3^/g. The pore size distribution for the SP-C was considerably wider, with a mean pore size of 2.5 nm and a pore volume of 0.4 cm^3^/g. As generally observed, amino-co-condensed particles do not have as well-ordered pore structure as pure silica particles [59]. The pore reduction of the SP-B might be explained by specific interaction between the phenyl groups and the polymer template during synthesis [63], thus leading to a shrinking in pore size [64,65]. The tubular pores in HNTs were significantly larger (10 nm) than the porous silica particles, but with lower pore volume, 0.2 cm^3^/g. All these characteristics are listed in Table 1.

### 3.2. Loading Capacity of the Volatile and the Non-Volatile Model Compounds in the Nanocarriers

**Figure 6:**
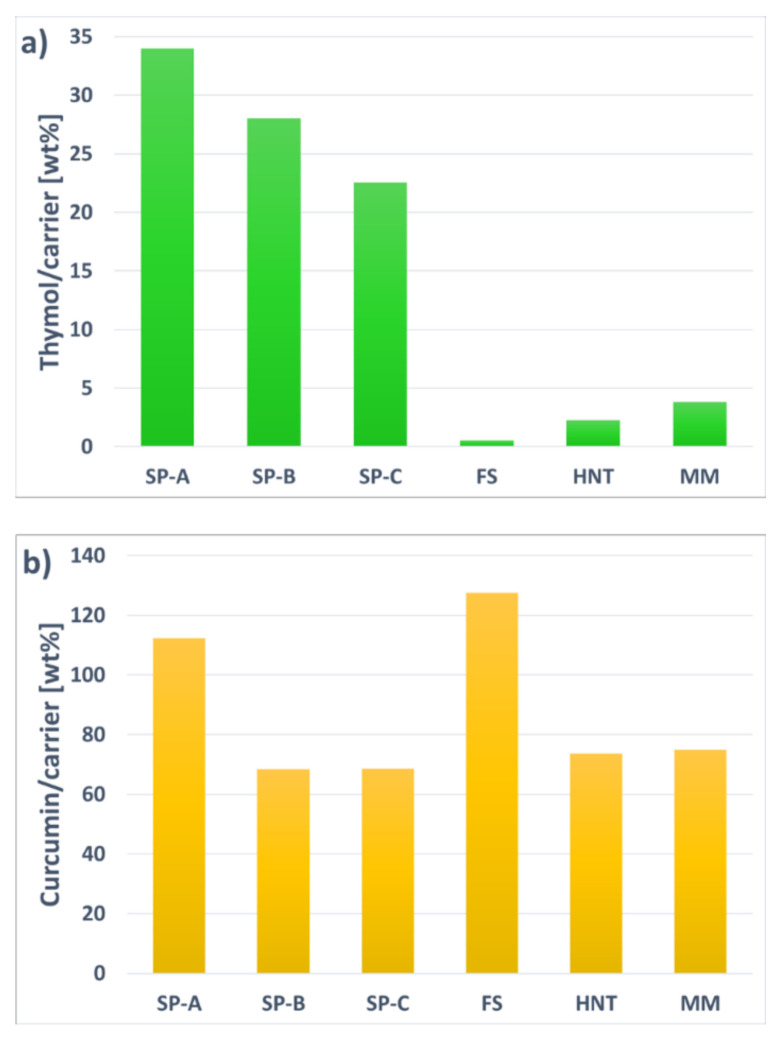
Loading degree of thymol (**a**) and curcumin (**b**) in the various carrier matrices.

The encapsulation efficiency of the nanocarriers was evaluated with two different compounds: thymol as a model for a volatile compound and curcumin as a model for a non-volatile compound. These compounds were chosen as they are widely characterized in terms of their antimicrobial and antioxidant properties. For comparison purposes, the loading procedure was identical for all different nanocarriers and active compounds. After the loading, the nanocarriers were washed and vacuum-dried to remove any active compound adsorbed onto the outer surface. The maximum amount of active compounds encapsulated by the nanocarriers is presented in Figure 6, and the results revealed a large difference in the loading capacity between the two distinct compounds. HNTs could not efficiently encapsulate the volatile thymol compound (Figure 6a), even though a high loading of another volatile compound, carvacrol, was reported earlier [27]. The reason for the low loading of thymol might be due to the washing and drying process, in which the open pore structure of the HNTs could not protect the thymol molecules. This was confirmed by the adsorption of thymol on FS and MM. Even though these materials had a high surface area (3–4 times higher than HNTs), they could not hold the volatile molecules due to the lack of a protective pore environment.

On the contrary, all the three porous silica particles seemed to be feasible encapsulation matrices for the volatile compound, even after removal of the compound adsorbed on the outer surface. Loading a volatile compound, in this case thymol, seemed to be highly dependent on the pore structure of the nanocarrier. The SP-A encapsulated the highest amount of thymol, 34 wt%, which was due to a high inner surface area and pore volume with a narrow mesoporous structure. The SP-B reached a slightly lower loading degree of 28 wt%. Even though these phenyl-co-condensed particles had the highest specific surface area, high pore volume, as well as a presumed compatible environment for accommodating the hydrophobic compound, thymol might not have been able to diffuse into the whole micropore structure. The SP-C had the lowest loading degree, 23 wt%, out of these three particle candidates. These particles had a lower specific surface area, and with the notion that they also were much smaller and thus exhibited a larger external surface, the inner surface was significantly decreased, which was also reflected in the lower pore volume. However, the larger pore size and broader pore size distribution of these particles seemed to be easily accessible for the thymol compound. The amino groups on the surface might also have positively influenced the absorption.

**Figure 7:**
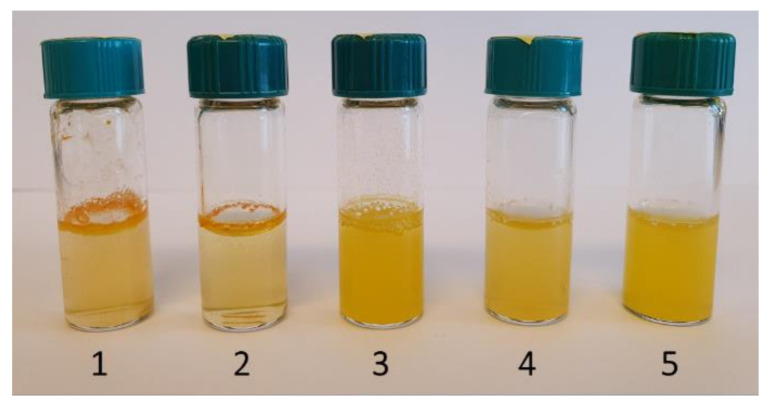
Solubility of free curcumin (bottle 1) and dispersibility of curcumin-loaded carriers (bottles 2–5) in water after slight shaking. Bottles 2–5: SP-A, PEG-coated SP-A, HNT, and PEG-coated HNT.

Similarly, when the highly hydrophobic curcumin molecules were loaded into the nanocarriers (Figure 6b), the difference in encapsulation efficiency between the SP-A and SP-B was even larger. This indicated that the curcumin molecules were not able to enter the micropores of the SP-B, and the curcumin was mostly adsorbed on the outer surface. For comparison purposes, the method of loading the curcumin was the same as for the thymol. However, the outer surface adsorbed curcumin was not considerably affected by the washing and drying steps, thus leading to a higher amount of curcumin adsorbed on the external surface. This was verified by loading curcumin onto the high external surface of FS, which resulted in the highest loading degree of all carrier candidates. However, the resultant highly hydrophobic curcumin-FS complex was not able to disperse in any aqueous solvent, as was the case for free curcumin (Figure 7). SP-C, HNTs, and MM all had lower surface areas, but more easily accessible pore structure. Especially HNTs have, despite their low surface area of only 5% compared to the surface area of the silica particles, reached a high loading degree, most probably due to the large easily accessible tubular pores. The positively charged inner surface of HNTs might also have affected the loading. Thus, SP-A and HNTs were concluded to have the most feasible pore environment for loading of the hydrophobic curcumin compound, and these two nanocarriers were therefore chosen as curcumin carriers for further experiments. To improve the dispersibility of these curcumin-loaded nanocarriers, a poly(ethylene glycol) (PEG) polymer was adsorbed on the surface (Figure 7). PEG is widely utilized for dispersing nanoparticles, especially in the biomedical field, by providing steric stabilization. Here, PEG had probably a synergistic effect on the dispersibility of the nanocarriers by both washing off curcumin adsorbed on the outer surface and coating of the particle surface. After PEG-coating, a decrease in loaded curcumin amount was observed for the SP-A, while HNTs did not lose any curcumin during the adsorption of PEG. This signified that curcumin was efficiently loaded into the tubular pores of HNTs and revealed the potential for HNTs as promising carriers, in terms of loading capacity, for hydrophobic non-volatile compounds such as curcumin.

### 3.3. Stability and Release of the Encapsulated Active Compounds

To further investigate the effect of the different nanocarriers on the stability and release of the active compounds, the number of nanocarriers loaded with active compound was narrowed down to only the most promising candidates. For curcumin we chose to compare the SP-A and HNTs, as mentioned above. For the thymol compound SP-A and SP-B were selected due to the high thymol-loading capacity of these particles, and the hydrophobic environment of SP-B, which could probably influence the stability and release of thymol.

**Figure 8:**
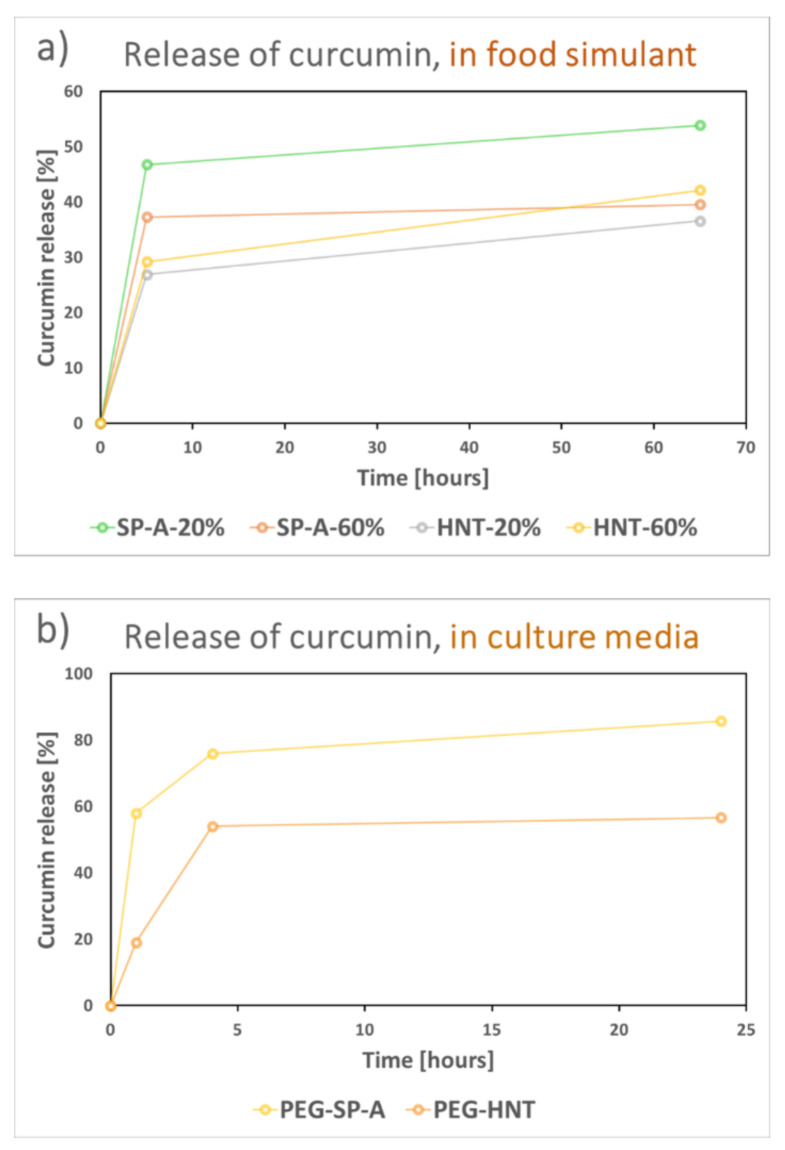
Release of curcumin from the SP-A and HNTs in (**a**) food simulant (20% ethanol) and (**b**) culture media (TSB with addition of 0.5 wt% Tween80).

For demonstration of the release from the curcumin-loaded nanocarriers, 20% ethanol was used as food simulant, in accordance with the Official journal of the European Union regulation on food simulants [57]. SP-A and HNTs were loaded with 20 wt% and 60 wt% curcumin to compare the possible differences in release due to loading amount, at the same time as comparing the release from the two distinct nanocarriers. The results are presented in Figure 8a and showed a higher burst release from the particles loaded with a lower amount of curcumin. These particles were less hydrophobic due to the lower curcumin amount, and the food simulant was probably thus more easily in contact with the curcumin in the pores. However, this was not seen for the HNTs, where curcumin might have been more packed in the tubular structure, rather than adsorbed on the high surface area of the particles, as also discussed above. Due to the solubility limit of curcumin, the release did not reach 100%. Normally a surfactant is utilized for demonstration of the release of such hydrophobic compounds. Earlier studies have reported the use of the cationic detergent cetrimonium bromide (CTAB) [66], the anionic surfactant sodium dodecyl sulfate (SDS) [67], or the nonionic polysorbate surfactant Tween80 up to 10 wt% [68] in the release media. When adding 10 wt% Tween80 to our release media, 100% of the curcumin was released within a few hours, demonstrating the possibility to alter the release rate by modifying the release media. For antimicrobial experiments we chose to add 0.5 wt% Tween80 to the culture media (Tryptic Soy Broth, TSB) so as not to affect the growth of the bacteria, and further utilized the PEG-coated nanocarriers for enhanced dispersibility. Prior to the antimicrobial tests, the curcumin release of the PEG-coated SP-A and HNTs was studied in culture media (TSB with 0.5 wt% Tween80), as shown in Figure 8b. The clear difference in burst release was also noted here. A lower overall release was also observed from the HNTs, probably due to a close-packing of hydrophobic curcumin molecules in the large tubular pores, which might have hindered the exposure of the molecules to the releasing media and thus the subsequent release.

**Figure 9:**
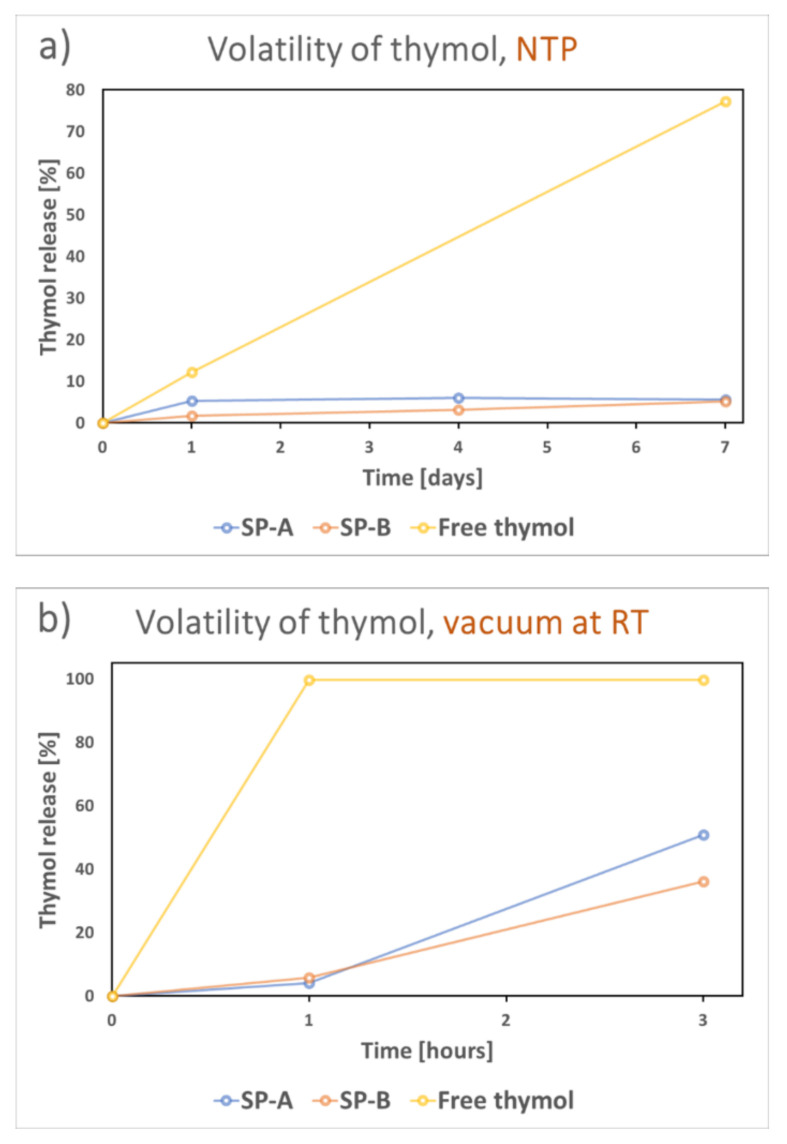
Comparison of volatility of free thymol crystals and encapsulated thymol open in (**a**) normal room temperature and pressure (NTP) and in (**b**) vacuum at room temperature (RT).

The thymol-loaded particles were studied in terms of both the stability and the release of the volatile thymol. Figure 9 presents the difference in volatility of free thymol and thymol loaded into SP-A and SP-B. In ambient conditions the particles protected the encapsulated thymol from volatilization (Figure 9a), while 80% of free thymol crystals volatilized during a period of seven days. This process was much faster under vacuum (Figure 9b), where all free thymol was lost after only one hour while the particles only lost about 5% of their loading. After 3 h of vacuum, the particles still had at least 50% of the loaded thymol remaining in their pores. A slight difference between SP-A and SP-B was noticed here, which might be due to the hydrophobic protective environment of SP-B. These results confirmed the volatility of thymol, and the probable instability of such a volatile compound if used, for example, for food packaging without a suitable carrier.

**Figure 10:**
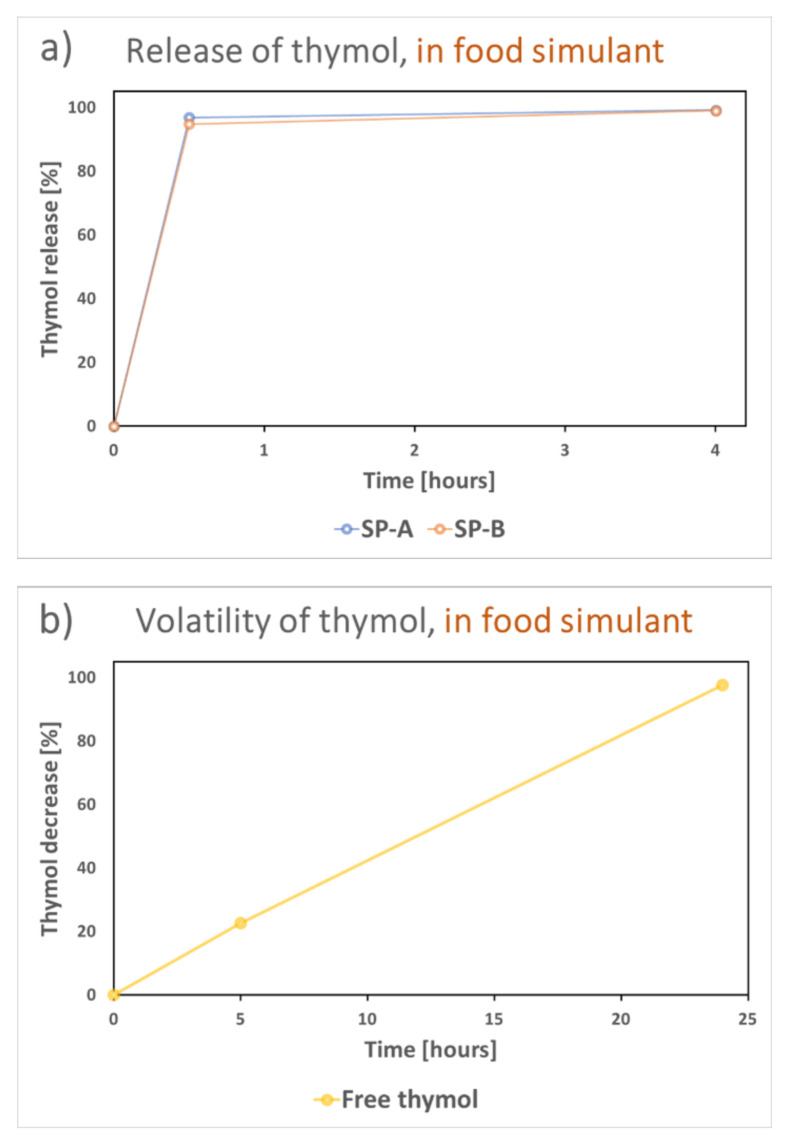
(**a**) Investigation of the release of thymol from the particles in the food simulant (20% ethanol), and (**b**) subsequent volatilization of thymol from the solvent.

Even though the thymol compound was stable inside the pore structure of the silica particles in the air, a rapid release occurred in the food simulant (20% ethanol), as shown in Figure 10a. This burst release of thymol from the particles in the food simulant demonstrated the potential drip-loss activation of the compound if incorporated into a food package. The free thymol released from the particles volatilized during a period of 24 h from the liquid phase, as demonstrated in Figure 10b.

### 3.4. Antimicrobial Response of the Loaded Nanocarriers

**Figure 11:**
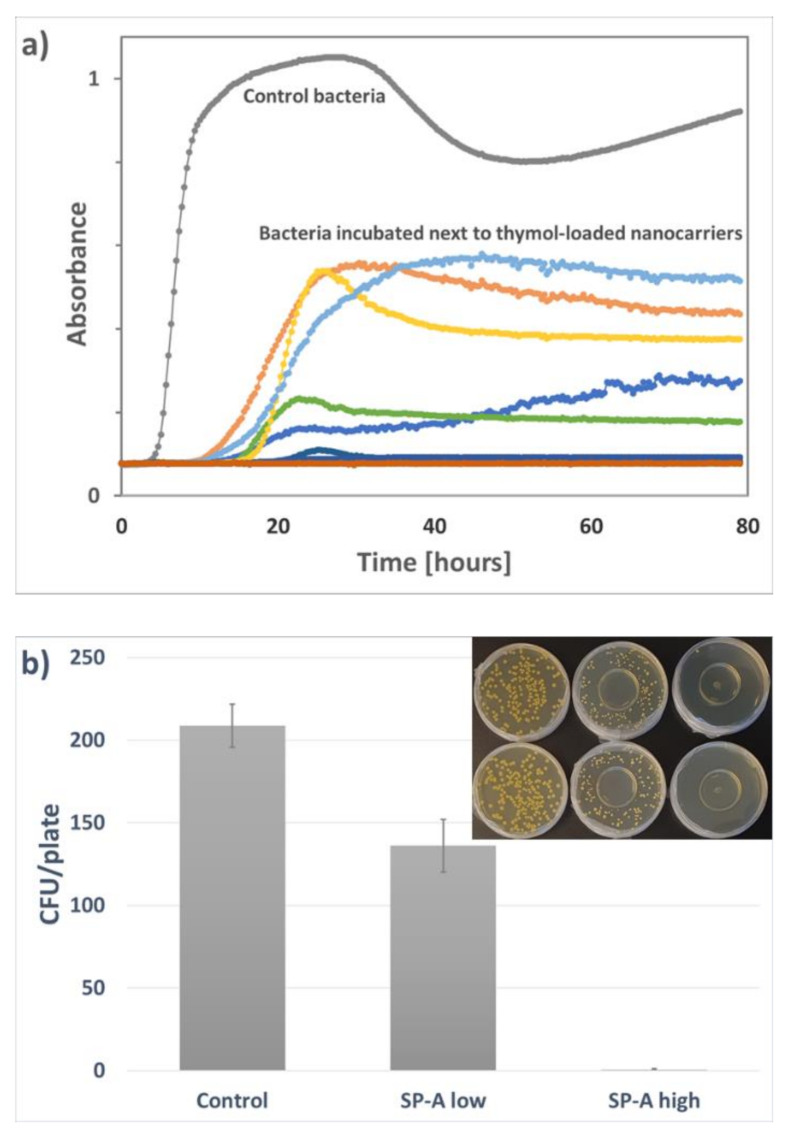
(**a**) Growth curves of *S. aureus* when incubated next to thymol-loaded SP-A up to 80 h. The growth curve for the control bacteria is an average of 20 samples. The treated samples (20 growth curves in total) vary significantly, with complete growth inhibition observed in 15 samples. (**b**) Growth of *S. aureus* (CFU/plate) after incubation with two concentrations of thymol-loaded SP-A dispersed in water (low: 100 µg/mL loaded thymol, and high: 300 µg/mL loaded thymol). Means and standard deviations from four parallels. The inset image represents two parallels from the experimental set-up.

The antimicrobial effect of the active compounds released from the nanocarriers was evaluated against *Staphylococcus aureus*, a Gram-positive bacterium very relevant for food safety. *S. aureus* is also well-known to be susceptible to natural antimicrobial compounds. The antimicrobial activity of the thymol-loaded nanocarrier was evaluated by comparing the growth curves of control bacteria and bacteria in close proximity to thymol-loaded nanocarriers dispersed in water. This time-to-detection method revealed a strong antimicrobial response of the released and volatilized thymol, with some variation from different parallels. From the total 20 parallel samples, severe inhibition was observed in five samples while no growth was detected in 15 samples within three days, as shown in Figure 11a. However, due to the probable leakage of thymol from the plates to the outer environment, the concentration of thymol might have varied significantly in the headspace over these cultures. This probably explains the observed variation of the treated samples. Thus, the concentration of thymol inside the plates could not be determined with this method. The vapor diffusion method was therefore chosen to compare the antimicrobial response at different concentrations of thymol-loaded nanocarriers dispersed in water, as presented in Figure 11b. A clear reduction in bacterial count was observed in the presence of 100 µg/mL loaded thymol (0.3 mg/mL SP-A loaded with 34 wt% thymol), and an almost total inhibition of bacteria was attained at a concentration of 300 µg/mL loaded thymol. The lower concentration of thymol had a local effect on the bacteria close to the thymol particles where the growth was inhibited or the size of the colonies reduced (Figure 11b, inset picture).

Despite earlier reports on the antimicrobial effect of curcumin on *S. aureus*, our efforts to study the antimicrobial effect of dissolved free curcumin by dilution method at concentrations up to 200 µg/mL did not succeed. Previous studies have reported minimum inhibitory concentrations (MICs) ranging from 125 to 250 µg/mL [69,70]. However, the MIC concentration might depend largely on the choice of method and the solvent used to dissolve curcumin. In the present study we used 10 wt% Tween80 to dissolve curcumin, with further dilution with TSB to a total Tween80 concentration of 0.5 wt%, the same as was used for the release study of curcumin (Figure 8b). Hence, the choice of solvent might have influenced the effect of free curcumin. Recent studies have also reported a much larger variation in sensitivity of different strains of pathogens toward curcumin than shown in previous reports [71], and discussed that the MIC concentrations, and thus the antimicrobial effect of curcumin, may vary significantly [72]. Consequently, in the present study it was not possible to study the antimicrobial effect of the curcumin released from the nanocarriers. Nevertheless, the loading and release studies performed here will still be valuable for further studies with other hydrophobic non-volatile antimicrobial compounds.

## 4. Conclusions

The present study compared and evaluated the loading efficiency of natural antimicrobial compounds into porous silica particles and nanoclays. The silica particles were designed to possess different pore structures and/or surface functionality. The results suggest that the nanocarrier must be optimized towards the specific compound of interest, to reach a high loading degree and further provide for a protective environment with subsequent release. For loading of the hydrophobic curcumin compound, which was here utilized as a model compound for a non-volatile substance, the tubular structured HNTs seem to be feasible carriers in terms of loading efficiency and dispersibility. However, the initial release in the food simulant of the hydrophobic compound was greatly affected by the pore structure, with a much higher burst release as well as overall release from the silica particles. On the contrary, loading of the volatile thymol compound seemed to be highly dependent on the pore structure of the nanocarrier. The large tubular pores of the HNTs could not provide a protective environment for the volatile thymol molecules, which resulted in a low loading degree. In contrast, a high loading was achieved in all the three different silica particles, with some variations depending on the pore size and inner surface area. It was demonstrated that the silica particles can protect the encapsulated thymol from volatilization under ambient conditions, as well as significantly slow down the volatilization under vacuum, compared to free thymol. Further, water-activated release of the encapsulated thymol was shown, which subsequently revealed great effectiveness in the vapor phase against *S. aureus*.

The results presented here confirm the feasibility of using inorganic nanocarriers to improve the functionality of natural active compounds for potential antimicrobial food packaging applications. However, for a successful utilization of a carrier system, the nanocarrier must be carefully chosen to meet the properties of the active compound to be encapsulated, as well as the desired outcome in the final application.

## Figures and Tables

**Table 1:** Characteristics of the nanocarriers.

Nano-Carrier	Morphology	Composition	Size (nm)	Zeta Potential (mV)	Specific Surface Area (m^2^/g)	Pore Volume (cm^3^/g)	Mean Pore Size (nm)	C-Value
**SP-A**	Porous spherical particles	Silica	1500	−27	720	0.8	2.1	62
**SP-B**	Porous spherical particles	Silica (co-condensation with phenylsilane)	1500	−22	830	0.7	1.5	160
**SP-C**	Porous spherical particles	Silica (co-condensation with aminosilane)	250	−19	420	0.4	2.5	35
**HNT**	Hollow tubes	1:1 (silica tetrahedral sheet, alumina sheet)	50 × 1000	−31	40 (64 *)	0.2	10.3	57
**MM**	Plate-shaped stacked layers	2:1 (silica tetrahedral sheet, alumina sheet)	Stacked nanolayers of various sizes	−21	220–270 *	X	X	X
**FS**	Particle-aggregates forming long branched chains	Silica	200-300*	−28	200 *	X	X	X

(*) reported values by the manufacturer; (X) data not available.

## Data Availability

The data presented in this study are available on request from the corresponding authors.
